# Social Prevalence Is Rationally Integrated in Belief Updating

**DOI:** 10.1162/opmi_a_00056

**Published:** 2022-07-01

**Authors:** Evan Orticio, Louis Martí, Celeste Kidd

**Affiliations:** Department of Psychology, University of California, Berkeley

**Keywords:** belief change, belief prevalence, misinformation, cue integration

## Abstract

People rely on social information to inform their beliefs. We ask whether and to what degree the perceived prevalence of a belief influences belief adoption. We present the results of two experiments that show how increases in a person’s estimated prevalence of a belief led to increased endorsement of said belief. Belief endorsement rose when impressions of the belief’s prevalence were increased and when initial beliefs were uncertain, as predicted by a Bayesian cue integration framework. Thus, people weigh social information rationally. An implication of these results is that social engagement metrics that prompt inflated prevalence estimates in users risk increasing the believability and adoption of viral misinformation posts.

## INTRODUCTION

The amount of data required to construct all of our beliefs “from scratch” is intractable given the limitations of our attention and the complexity of the world. Additionally, most truths cannot be determined by direct reference to the physical world anyway (Festinger, 1954). Instead, relevant evidence remains inaccessible to the individual for most real-world beliefs and must be mediated through other agents (Perfors & Navarro, 2019). Belief formation thus depends on information sampled from the social world. This process is distinct from normative social influence, in which conformity to a group norm takes precedence over conflicting private information about the ground truth (Deutsch & Gerard, 1955). Rather, this social sampling is necessary for acquiring information that is complex or otherwise unavailable to the learner.

Empirical evidence makes clear that social information affects beliefs, even while it leaves open how and why specific social information is integrated. This work dates back to Asch (1951), who demonstrated that people can report erroneous perceptual judgments when influenced by inaccurate information from confederates. Likewise, appeals to expert consensus effectively promote belief change, for example, for anthropogenic climate change (Goldberg et al., 2020; van der Linden et al., 2019; van der Linden, Leiserowitz, et al., 2015), nuclear power (Kobayashi, 2018), GMOs (Kerr & Wilson, 2018), and vaccination (van der Linden, Clarke, & Maibach, 2015). These social influences could be the result of social pressures to conform to others, especially those of higher status. Van der Linden et al.’s Gateway Belief Model (2019) argues instead that social information simply *licenses* belief revision. Alternatively, a rational account would predict social information should be integrated with the person’s a priori beliefs in ways that are sensitive to the reliability of both information sources.

Evidence in support of rational accounts of social information integration includes that individuals find a larger number of people more convincing than a smaller number (e.g., Bond, 2005). Relatedly, the number of endorsers of a belief is known to be a key influencer of belief adoption. For example, Ransom et al. (2021) found that the raw number of people endorsing a view was a more important predictor of belief adoption than the diversity of arguments made. Some evolutionary models of social learning also have a rational flavor, suggesting intelligent organisms sometimes learn socially by adopting a copy-when-uncertain strategy. Learners copy the behavior of others when they have no relevant private information (Kendal et al., 2009; Rendell et al., 2010; Toelch et al., 2014). This evidence is consistent with the rational uptake of social information into one’s behaviors, but examines cases of discrete choice rather than graded integration of new social information with held beliefs.

### Informational Social Influence as Cue Integration

The role of social information in belief formation may be better understood within a cue integration framework, borrowed from the perception literature (e.g., Ernst & Banks, 2002; Knill & Richards, 1996). Observers combine signals from different perceptual senses to arrive at a unified percept. In forming a new belief, a learner similarly integrates private information, like domain-relevant knowledge, with social information, like the prevalence of a belief in a sampled population. Importantly, each cue is flexibly weighted according to its estimated reliability.

Strong reliance on social information can be rational, especially under conditions of uncertainty. Within this framework, even the conformity to the majority in Asch’s (1951) line-matching experiments could reflect optimal information integration rather than—or in addition to—normative pressure to conform. If the participant assumes that others are unbiased and have low error rates, as would reasonably be expected in simple perceptual tasks, then the probability of the participant being correct given the confederates’ convergence on a different answer is low (Toelch & Dolan, 2015). Thus, an optimal Bayesian learner would give high weight to the social information.

### The Role of Social Information in Misinformed Belief

These principles may also explain viral cases of online misinformation. Much of the existing literature on conspiracy and pseudoscientific beliefs has sought to explain them away by appealing to ancillary influences on belief formation, like emotionality (Vlasceanu et al., 2020), political extremism (Van Prooijen et al., 2015), inattentiveness (Pennycook & Rand, 2019), and bullshit receptivity (Pennycook & Rand, 2020). However, these arguments fail to acknowledge that many online platforms have created an environment that makes rational belief formation processes highly susceptible to misinformation. False news has been found to spread “farther, faster, deeper, and more broadly” than true news online (Vosoughi et al., 2018, p. 1150), due in part to the formation of echo chambers. Given the high-virality potential of online misinformation, it is likely that people encounter high social engagement metrics, like the number of likes and shares, accompanying false claims. These metrics may serve as a cue to a belief’s prevalence in the population, which may in turn impact the user’s own impression of the belief’s legitimacy. This information may be particularly problematic when it is encountered while a person is “doing their own research” and thus in a state of high uncertainty.

### Overview of Present Research

We conduct two experiments to test whether increasing the perceived social prevalence of a belief increases its believability in the absence of direct evidence. Experiment 1 tests this hypothesis using a set of current, real-world conspiratorial and pseudoscientific beliefs. We further test whether prevalence information is integrated rationally as predicted by a cue integration framework. If so, the social prevalence cue should be weighted against existing evidence such that it elicits the strongest belief change (a) when new data is most convincing, that is, estimates of the belief’s prevalence change the most in response to the data, and (b) when the initial belief is most uncertain. Experiment 2 replicates Experiment 1 but implements a cover task to reduce possible demand characteristics. In the next section, we present the methods for each experiment and then discuss the results of both together.

Understanding how social prevalence estimates impact belief adoption is important for understanding how to develop interventions against echo chambers online. The ubiquity and prominence of inflated cues to the social prevalence of beliefs (e.g., via visible metrics of likes, shares, or views) may increase engagement while at the substantial cost of leading people to be more likely to adopt beliefs they would not under less artificial circumstances.

## EXPERIMENT 1

Participants read a series of statements relating to real-world conspiratorial, pseudoscientific, or misinformed beliefs (e.g., “The earth is flat,” “Wearing masks is harmful to the health of the mask wearer,” “The U.S. government planned the 9/11 attack on the World Trade Center”; see the Supplemental Materials for the full list). We chose beliefs that are recently attested but thinly and weakly held (Martí et al., 2021). These low-probability beliefs serve as a stringent test of the strength of social influence. On each trial, participants provided (1) a likelihood estimate of the belief (“How likely do you think it is that the statement is true?” on a 0–100 slider scale), and (2) a prevalence estimate (“How many people out of 100 do you think believe the statement is true?”). The experiment began with three simple practice trials involving statements of fact (e.g., “Plants need water to grow”) with feedback. We included four more trials of the same type in the main task without feedback as attention checks, for a total of 37 trials.

**Figure 1.**
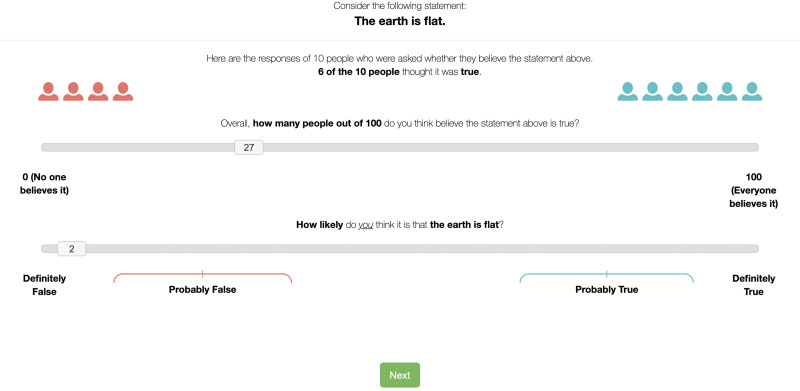
Example trial from block 2.

Participants repeated the same trials in a shuffled order in a second block. However, before evaluating the likelihood and prevalence of each statement, participants viewed a sample of data indicating how many of 10 survey respondents believed the shown sentence (see Figure 1). The sample for the 10 people approximately matched the participant’s own estimate for the prevalence of the belief in block 1 (Control condition) on half of the trials. On the other half of trials, the number of people in the sample endorsing the pseudoscientific or conspiratorial belief was 40% higher than the participant’s initial prevalence estimate (Higher Prevalence condition). For example, if a participant estimated in block 1 that 19 of 100 people believe the given statement is true, then in block 2 they would be shown that 2 of the 10 survey respondents believe the statement (Control condition) or 6 of the 10 believe it (Higher Prevalence condition). Note that, for variation, some statements were related to an attested conspiracy or pseudoscientific belief but worded in their inverse (true) forms, for example, “Humans have landed on the moon.” In this case, the prevalence of the sample was 40% lower, that is, in the direction of the empirically unsupported belief, in the Higher Prevalence condition. These items are reverse coded in our analyses, so we retain the Higher Prevalence label for simplicity. The participants then gave estimates for the prevalence and likelihood of the belief.

We debriefed participants at the end of the experiment. We provided a representative prevalence estimate of all of the beliefs based on a large sample of over 900 Americans (Martí et al., 2021) and reminded participants that prevalent beliefs are not necessarily true.

Experiment 1 guarded against the risk of participants feeling pragmatic pressure to change their prevalence and belief estimates by nature of being asked twice (e.g., demand characteristics) in two ways. First, we collected ratings for a large number of statements (30) in shuffled orders to make remembering what had been asked previously difficult. Second, we asked participants after the experiment what they believed the experiment to be about in order to determine whether participants guessed the purpose and may have altered their responses accordingly. Experiment 2 adds the additional guard of a cover task.

## EXPERIMENT 2

Experiment 2 replicates Experiment 1 with one change: before block 2, participants were told that the prevalence data was part of a memory task. This cover task was intended to provide an additional guard against demand characteristics. We instructed participants to memorize how many of the 10 survey respondents believed each statement in preparation for a memory quiz.

Similar levels of belief change across the two experiments would suggest that any observed belief change is not driven by demand effects. Participants’ responses in a debriefing survey provide further evidence against the role of demand effects (see the Supplemental Materials).

### Participants

We recruited 608 (*N* = 403 in Experiment 1; 205 in Experiment 2) American adults fluent in English through Prolific (www.prolific.co) to participate in an 18-min online experiment. Only American adults participated because some experimental items referenced U.S.-specific cultural beliefs. We compensated participants at a rate of $10/hour, with an opportunity to receive a bonus for good performance. We obtained informed consent from all participants and used methods approved by the University of California, Berkeley Institutional Review Board. We preregistered both experiments prior to data collection at https://aspredicted.org/sy9wv.pdf (Experiment 1) and https://aspredicted.org/vc8jr.pdf (Experiment 2).

## RESULTS

### Exclusions

#### Experiment 1.

Eighteen participants were excluded for failing more than one of four attention checks, and an additional 19 participants were excluded for giving blank or unreasonable responses to one or more of six bot-catch questions (e.g., “What’s your favorite frozen treat?”) intermixed between experimental trials. These subject-level exclusions resulted in a final sample of 366.

In addition to these preregistered exclusions, we also excluded trials in which participants initially endorsed the empirically unsupported belief with a likelihood rating of over 60%, because the 40% exaggeration in the Higher Prevalence condition could not be applied to these. These trials constituted 13.1% of our data, and their removal did not significantly affect the interpretation of any analyses (see the Supplemental Materials).

#### Experiment 2.

Three participants were excluded for failing more than one of four attention checks. No additional participants were excluded, yielding a final sample of 202 participants. Trial-level exclusions (14.6% of all trials) were made using the same criteria as Experiment 1, as preregistered.

### Participants Revise Their Estimates of Prevalence in Light of Prevalence Data

#### Experiment 1.

If our manipulation worked as intended, participants’ estimates of the prevalence of these beliefs should have increased after seeing the Higher Prevalence data, but remained the same in the Control condition. On average, participants’ prevalence estimates increased by 21.0% in the Higher Prevalence condition and remained relatively stable in the Control condition (decreased by 1.7%). A *t* test indicates that this difference is statistically significant (95% CI = [22.1, 23.4]; *t*(6904.9) = 65.75, *p* < .001, *d* = 1.35), confirming the effectiveness of the manipulation.

#### Experiment 2.

Participants’ prevalence estimates increased by 25.6% in the Higher Prevalence condition and marginally decreased by 1.0% in the Control condition. A *t* test indicates that this difference is statistically significant (95% CI = [25.8, 27.5]; *t*(3991.8) = 61.59, *p* < .001, *d* = 1.71).

### Participants Revise Their Beliefs in Line With New Prevalence Information

#### Experiment 1.

**Figure 2.**
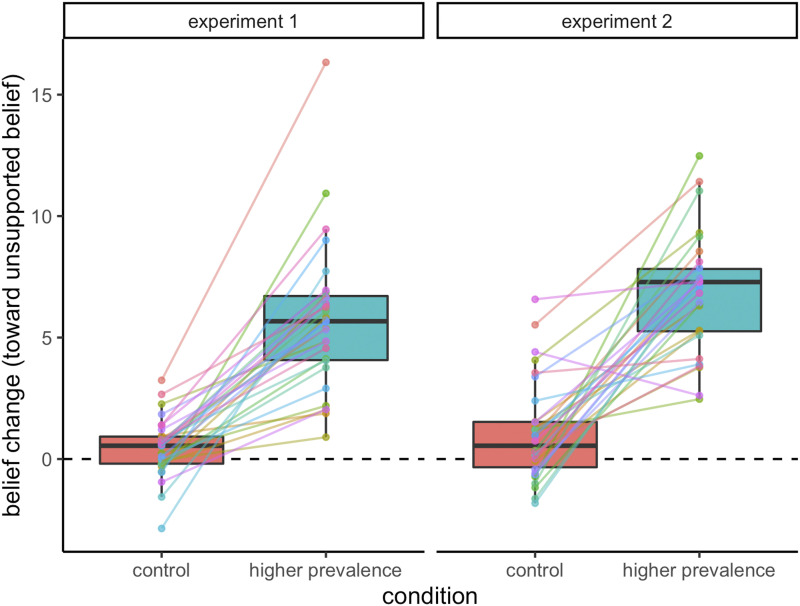
**Boxplot of belief change after seeing a sample of data that either matched participants’ expectations (Control) or indicated a higher prevalence of belief (Higher Prevalence) for Experiments 1 and 2.** Points represent the mean belief change per item, with lines showing the effect of condition for each item.

Our main prediction was that participants would increase their endorsement of a belief when given data indicating that the belief is more prevalent than they expect. Figure 2 shows the mean amount of belief change per item in each condition for both experiments (for a breakdown by item, see the Supplemental Materials). As predicted, participants’ ratings of the likelihood of these beliefs increased by a mean of 5.44% in the Higher Prevalence condition and remained relatively stable (0.46% increase) in the Control condition (95% CI = [4.43, 5.52]; *t*(9046.5) = 17.82, *p* < .001, *d* = 0.365), indicating that exposure to samples with higher belief prevalence influences the plausibility of the belief.

#### Experiment 2.

The main effect of condition was replicated. Participants’ likelihood ratings increased by a mean of 6.59% in the Higher Prevalence condition, compared to an increase of 1.22% in the Control condition (95% CI = [4.59, 6.15]; *t*(5049.1) = 13.50, *p* < .001, *d* = 0.375).

### Belief Change Is Commensurate With Change in Prevalence Estimate

#### Experiment 1.

**Figure 3.**
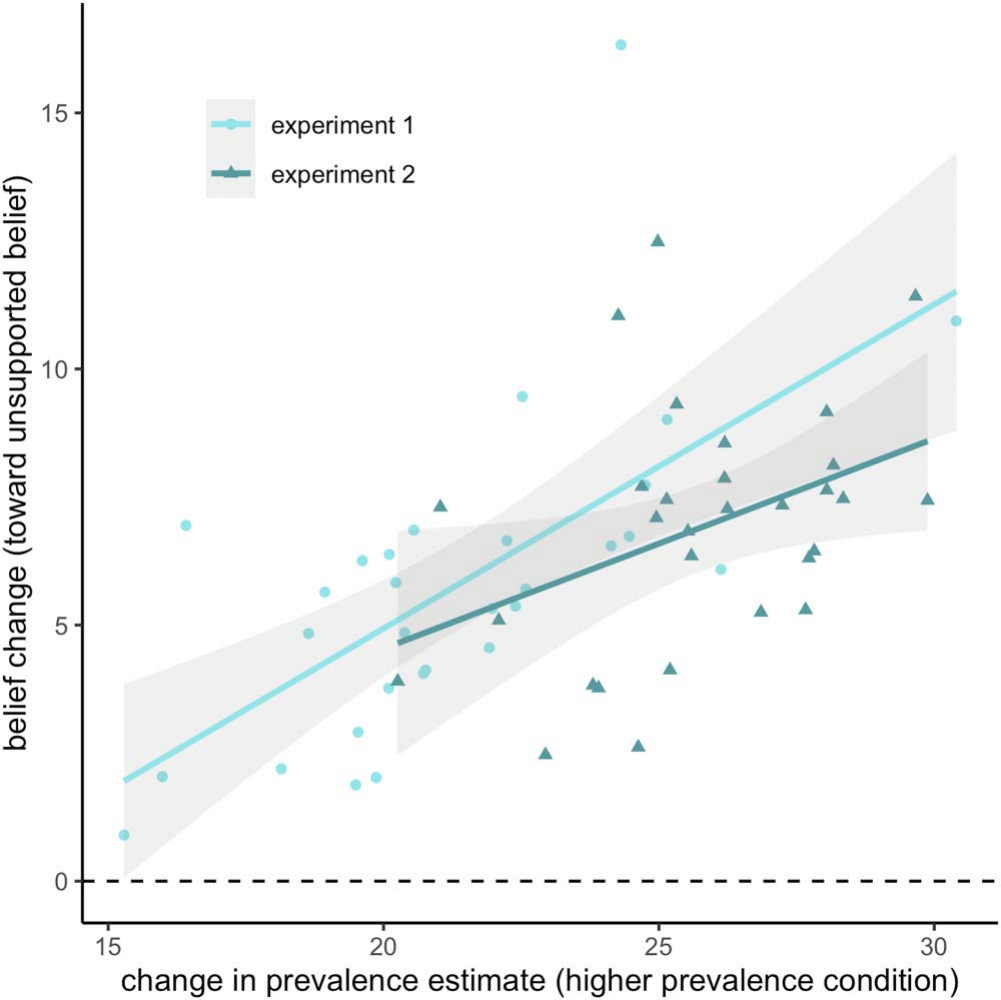
**Mean belief change vs. mean change in prevalence estimate for each item in the Higher Prevalence conditions of Experiments 1 and 2.** Lines represent the fit from linear regression for each dataset with 95% confidence intervals.

If social prevalence is treated as an independent source of information that is integrated rationally with prior beliefs, then greater changes in one’s estimation of the prevalence of a belief should result in stronger belief updating. That is, a stronger prevalence cue should carry more weight and have a stronger effect on the ultimate belief. Figure 3 shows the relationship between change in prevalence estimate and resulting belief change within the Higher Prevalence condition of both experiments. We ran a linear mixed-effects model predicting belief change with condition and change in prevalence estimate as fixed effects and random intercepts per participant and item. This model revealed significant main effects of condition (β = 1.29, *t* = 3.91, *p* < .001) and change in prevalence estimate (β = 0.16, *t* = 9.01, *p* < .001), suggesting that larger increases in prevalence estimates of a belief led to larger increases in personal belief endorsement. There was no significant interaction (*p* = .81). The model accounted for 19.0% of the variance in belief change (conditional *R*^2^).

#### Experiment 2.

A linear mixed-effects model with an identical structure revealed only a significant main effect of change in prevalence estimate (β = 0.16, *t* = 6.05, *p* < .001). Unlike in Experiment 1, the main effect of condition was not significant after controlling for change in prevalence estimate (*p* = .16). The model accounted for 16.4% of the variance in belief change.

### Belief Change Is Dependent on Initial Certainty

#### Experiment 1.

**Figure 4.**
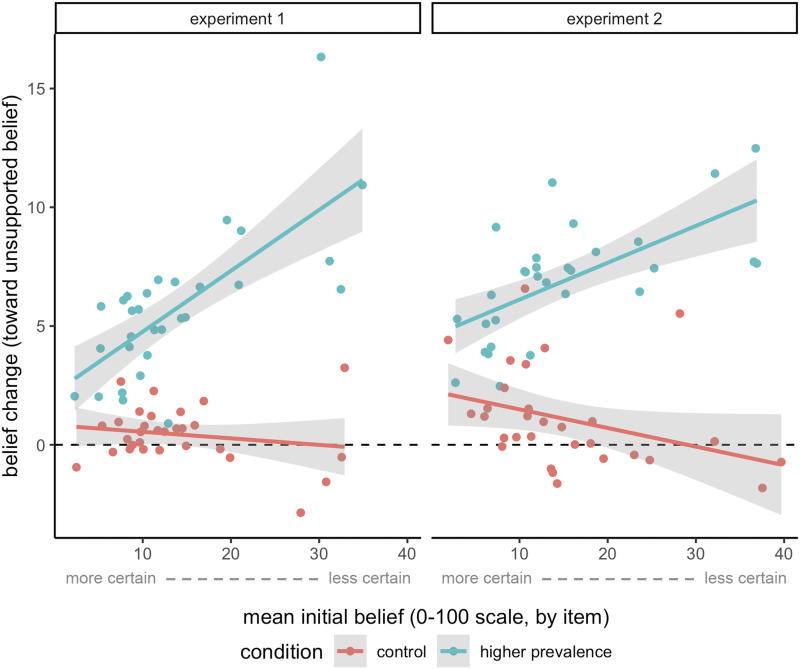
**Mean belief change vs. mean initial belief for each item, by condition.** Lines represent the fit from linear regression for each condition, with 95% confidence intervals. As initial belief increases toward 50% likelihood, certainty decreases. Belief change is plotted directly against certainty in the Supplemental Materials.

We predicted that rational belief updating should also depend directly on the initial certainty of the belief. While novel evidence about social prevalence should bear significant weight under conditions of uncertainty, high-certainty beliefs should be relatively resistant to change regardless of social prevalence. Figure 4 illustrates the relationship between initial belief (directly related to certainty) and belief change across prevalence conditions. We operationalize certainty as the absolute distance from 50% on the likelihood scale. We fit a linear mixed-effects model with standardized certainty and prevalence condition as fixed effects and random intercepts per participant and item. The model revealed main effects of both scaled certainty (β = 2.42, *t* = 11.91, *p* < .001) and prevalence condition (β = 4.93, *t* = 18.91, *p* < .001), as well as a significant interaction (β = −2.84, *t* = −10.76, *p* < .001). The model accounted for 18.1% of the variance in belief change. Only in the Higher Prevalence condition, where belief change was motivated by data, lower levels of certainty predicted higher belief change as hypothesized.

We replicated our predicted effect of certainty with an additional linear mixed-effects model using change in prevalence estimate as a continuous predictor instead of the dichotomous condition variable. This model had an otherwise identical structure to the first. Again, main effects of standardized certainty (β = 1.33, *t* = 8.69, *p* < .001) and change in prevalence estimate (β = 0.17, *t* = 25.59, *p* < .001) were significant. Crucially, the same significant interaction was observed (β = −1.92, *t* = −14.99, *p* < .001), such that with higher changes in prevalence estimates, initial certainty of a belief negatively predicted belief change. This model accounted for 23.7% of the variance in belief change.

The positive relationship between certainty and belief change in the Control condition is not predicted by rational accounts of belief change and likely stems from an inherent property of our experimental design. Participants’ estimates of prevalence were more likely to differ more from their own initial beliefs when their own initial beliefs were more certain. This pattern is consistent with weak priors resulting in a person reverting to their knowledge of social prevalence when making up their mind. Therefore, the difference between the data shown and the participant’s own initial belief was higher under conditions of high certainty. To control for this confound, we added the raw difference between the participant’s initial prevalence estimate and their own initial belief as a fixed effect to the previous model, and used it to predict belief change in the Control condition. This model revealed that, after controlling for the confound, the unpredicted positive effect of certainty in the Control condition disappeared (*p* = .28). The predicted effects remained: prevalence change (β = 0.21, *t* = 13.15, *p* < .001), the difference between initial prevalence estimate and initial belief (β = 3.85, *t* = 19.22, *p* < .001), and the negative interaction between certainty and prevalence change (β = −1.92, *t* = −12.18, *p* < .001) all stayed significant. The model accounted for 25.7% of the variance in belief change. The negative interaction suggests that certainty also drove belief change in a rational manner in the Control condition when changes in prevalence estimates were sufficiently high.

#### Experiment 2.

Identical linear mixed-effects models replicated all of the effects from Experiment 1. The first model using prevalence condition as a predictor revealed a significant main effect of scaled certainty (β = 2.48, *t* = 8.59, *p* < .001), a significant main effect of prevalence condition (β = 5.45, *t* = 14.58, *p* < .001), and a significant negative interaction (β = −3.75, *t* = −9.90, *p* < .001). The model accounted for 16.5% of the variance in belief change. The second model using prevalence change as a predictor revealed a significant main effect of scaled certainty (β = 0.87, *t* = 4.09, *p* < .001), a significant main effect of prevalence change (β = 0.19, *t* = 19.37, *p* < .001), and a significant negative interaction (β = −1.96, *t* = −10.33, *p* < .001). This model accounted for 18.9% of belief change. As before, after controlling for the confound, the unpredicted positive effect of scaled certainty became nonsignificant (*p* = .25). Also as before, predicted effects in the model still replicated: we observed a main effect of prevalence change (β = 0.22, *t* = 9.03, *p* < .001), a main effect of the difference between initial prevalence estimate and initial belief (β = 3.65, *t* = 11.89, *p* < .001), and a negative certainty by prevalence change interaction (β = −1.02, *t* = −4.21, *p* < .001). This model accounted for 18.6% of belief change.

## GENERAL DISCUSSION

In two experiments, we demonstrated that increasing people’s perceptions of the general prevalence of a belief can cause them to endorse that belief more strongly, devoid of any direct evidence. Participants were presented with new prevalence data from an anonymous 10-person sample that conflicted with their prior about a belief’s prevalence by a uniform amount. This social prevalence clue alone was enough to inspire belief change, despite the fact that all items pertained to uncommon, empirically unsupported beliefs from a variety of real-world domains. Additionally, belief change conformed to two key predictions of a Bayesian cue integration framework. First, belief change was sensitive to the reliability of the new prevalence cue. When the new prevalence data was deemed more reliable, as indexed by larger changes in prevalence estimates, belief change increased. Second, belief change was governed by the initial certainty of the participant’s belief, with more weakly held beliefs changing more dramatically. These results were replicated in Experiment 2, thus ruling out the role of demand characteristics. Taken together, these findings suggest that decontextualized, nonauthority social prevalence information serves as a cue that people rationally integrate with existing private evidence according to the relative reliability of both information sources.

It is important to note that participants in our experiment were not simply blindly updating their beliefs to match the prevalence information that they were shown. Recall that the prevalence data shown in the Control condition matched participants’ initial estimates of the broader prevalence of a belief, and not their personal ratings of its likelihood. Participants were aware of this distinction; their initial prevalence estimates differed from their own initial likelihood estimates by a mean of 16.5% across experiments. Thus, before encountering the prevalence manipulation, participants demonstrated an implicit understanding that their belief may not be representative of that of the broader population. Further, prevalence data in the Higher Prevalence condition was 40% higher than the participant expected, yet participants’ final prevalence estimates changed by a mean of only 22.6%. Thus, participants did not uncritically trust the prevalence data, but rather integrated it with their existing belief about the prevalence of each claim.

Further evidence of this rational integration is that changes in prevalence estimates did not always lead to changes in private endorsement of the belief. For example, in Figure 4, we see that people do not update their beliefs when they are held with high certainty. A cue integration framework also predicts cases in which consensus information does not motivate belief change. Instead, the prevalence cue is evaluated against the strength of one’s prior belief. The possibility of bias or error in social evidence may be particularly salient in domains with widespread ignorance or strong political polarization (Lees & Cikara, 2021). There is evidence that people are able to correct for such bias. Learners rationally discount the weight of a group’s testimony when their source is shared and thus statistically dependent (Whalen et al., 2018; but cf. Yousif et al., 2019). Sensitivity to possible selection bias also can lead people to paradoxically draw weaker conclusions from stronger evidence when the evidence is presented by a social agent (Perfors et al., 2018). Future work should investigate cues people use to judge the reliability of prevalence data in paradigms like ours.

### Implications for Countering False Beliefs Online

Our findings have direct implications for interventions against the spread of misinformation online. We found that inferring a belief is more prevalent increases the likelihood of belief adoption; thus, high-engagement metrics attached to viral misinformation posts may promote stronger adoption of misinformed beliefs online. This could explain why people rate news from low-credibility sources as higher quality when engagement metrics are present (Chung, 2017). Further, high-engagement metrics elicit more sharing and less fact-checking from users in a simulated social media feed (Avram et al., 2020). This is of particular concern for viral misinformation posts, which can bear engagement numbers in the hundreds or thousands, as opposed to our experimental manipulation with data on 10 people. Hiding social engagement metrics entirely for posts relaying false or misleading information may therefore help reduce false belief. Future work should test interventions along these lines in an effort to counter the online misinformation crisis.

## ACKNOWLEDGMENTS

We thank Steve Piantadosi, Holly Palmeri, Carolyn Baer, members of the Kidd Lab, and anonymous reviewers for constructive feedback on this work.

## FUNDING INFORMATION

CK, Hellman Fellows Fund. CK, Defense Advanced Research Projects Agency (US), Award ID: HR001119S0005. CK, Berkeley Center for New Media.

## AUTHOR CONTRIBUTIONS

EO: Conceptualization: Equal; Formal analysis: Lead; Methodology: Lead; Visualization: Lead; Writing – original draft: Lead; Writing – review & editing: Lead. LM: Methodology: Supporting; Resources: Lead. CK: Conceptualization: Equal; Formal analysis: Supporting; Funding acquisition: Lead; Methodology: Supporting; Supervision: Lead; Visualization: Supporting; Writing – original draft: Supporting; Writing – review & editing: Supporting.
