# Supplementary material for: Social Prevalence Is Rationally Integrated in Belief Updating
Source: Open Mind (Camb). 2022 Jul 1;6:77–87. doi: 10.1162/opmi_a_00056 (PMC9692052; doi:10.1162/opmi_a_00056)

Supplemental Materials.

A. Experimental Items Ranked by Size of Main Effect Across Experiments 1 and 2

	Statement	Main Effect	Higher Prevalence	Control
1	Handwriting analysis can reveal aspects of the writer's personality or mental state	12.05	11.46	-0.58
2	Accused child rapist Jeffrey Epstein killed himself in solitary confinement awaiting trial	10.57	14.81	4.25
3	John F Kennedy's assassination was a coordinated effort by multiple people	9.67	7.72	-1.95
4	Most physical ailments can be remedied through realignment of the skeletal system	9.03	8.46	-0.58
5	Hypnosis can be used to uncover repressed memories	8.50	6.87	-1.63
6	Hospitals are inflating the number of COVID-19 deaths	8.25	8.30	0.05
7	Under the same exposure conditions a black person is more likely to contract COVID-19 than an equally healthy white person	8.07	8.98	0.92
8	Autism is caused by environmental toxins	7.05	6.96	-0.10
9	HIV causes AIDS	6.65	7.11	0.46
10	Powerful people are suppressing known effective treatments for COVID-19	6.63	6.91	0.27
11	The US government planned the 9/11 attack on the World Trade Center	5.89	6.54	0.65
12	Humans and dinosaurs coexisted on the earth	5.72	4.95	-0.76
13	Aliens are currently visiting the earth	5.64	6.51	0.87
14	NIH Director Anthony Fauci is misleading people about COVID-19 because he stands to gain financially from a vaccine	5.48	6.17	0.69
15	Hillary Clinton was involved in a child sex-trafficking ring centered around a pizza restaurant in Washington DC	5.47	4.96	-0.52
16	Children can get or spread COVID-19	5.36	5.64	0.28
17	People can catch COVID-19 outdoors	5.13	6.45	1.32
18	People can maintain consciousness after death and visit living people	4.65	5.70	1.04
19	Powerful people planned the release of the COVID-19 virus	4.28	5.60	1.33
20	The media has exaggerated COVID-19 risks to undermine Donald Trump's presidency	3.92	7.09	3.16
21	Vaccines cause autism	3.67	4.29	0.62
22	Humans have landed on the moon	3.48	4.42	0.94
23	COVID-19 is more deadly than the flu	3.43	6.38	2.95
24	Cancer can be cured through prayer alone	3.43	3.28	-0.15
25	Wearing masks is harmful to the health of the mask wearer	2.53	5.51	2.98
26	Masks reduce the likelihood of transmission of COVID-19	2.44	3.27	0.83
27	Bill Gates is planning on tracking people with microchips using COVID-19 testing and vaccination	2.28	3.44	1.16
28	Climate change is caused by humans	1.94	2.03	0.09
29	Donald Trump has been working to stop an elite group of Satan-worshipping pedophiles	1.69	2.29	0.60
30	The earth is flat	1.27	2.25	0.97

B. Boxplot of Belief Change by Item (aggregated across Experiments 1 and 2)

Boxplot of belief change in each condition broken down by item. Data is aggregated across Experiments 1 and 2. Points represent condition means.

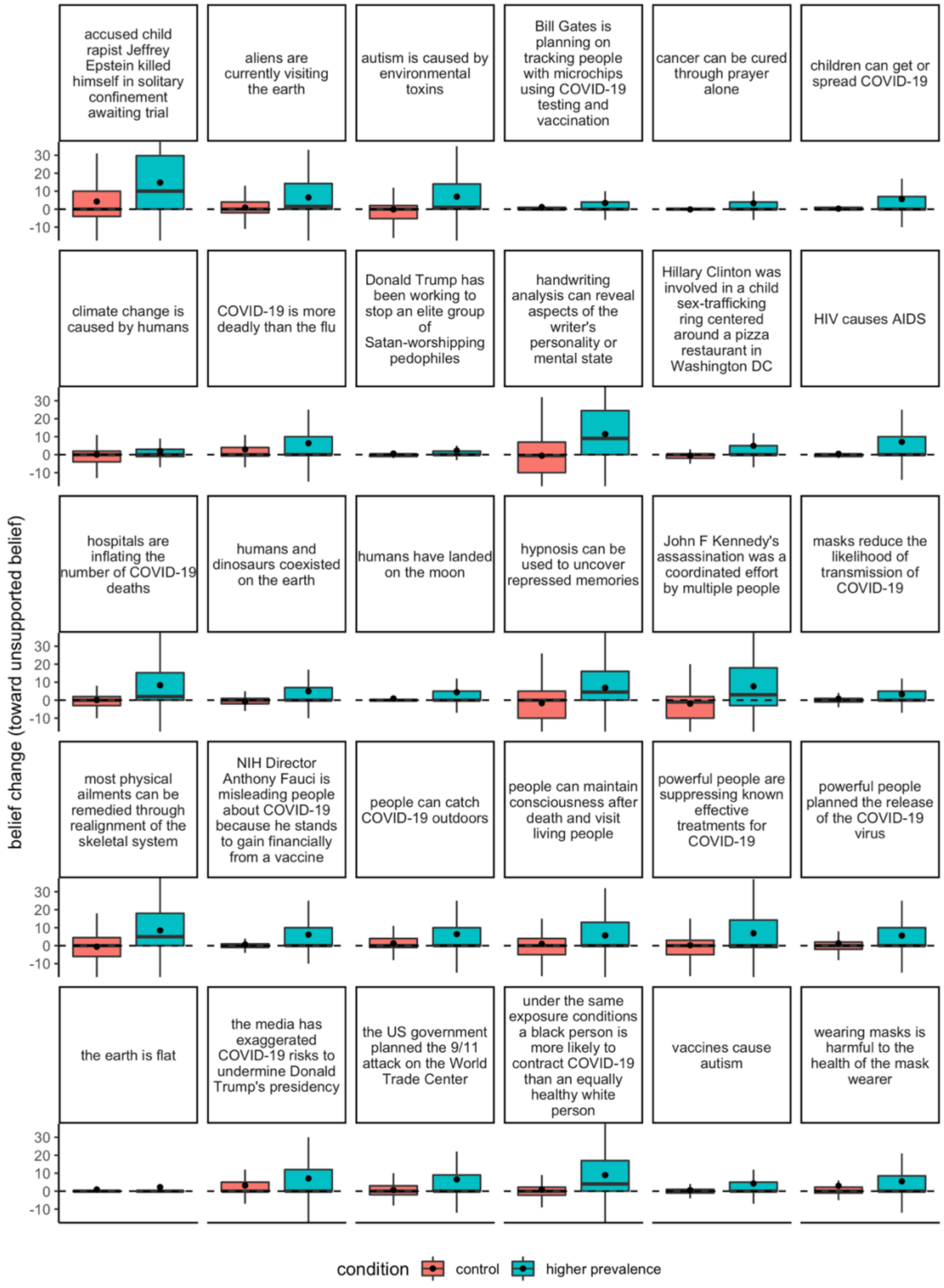

C. Results of Experiment 1 Without Trial-Level Exclusions

The following are the results of all reported analyses without the non-preregistered trial exclusions.

i. Participants revise their estimates of prevalence in light of prevalence data

On average, participants' prevalence estimates increased by 21.2% in the Higher Prevalence condition and remained relatively stable in the Control condition (decreased by 1.3%). A paired t-test indicates that this difference is statistically significant (95% CI = [21.9, 23.1]; $t(5489) = 73.22$, $p < 0.001$, $d = 0.99$).

ii. Participants revise their beliefs in line with new prevalence information

As predicted, participants' ratings of the likelihood of these beliefs increased by a mean of 5.19% in the Higher Prevalence condition and remained relatively stable (decreased by 0.32%) in the Control condition (95% CI = [4.97, 6.04]; $t(5489) = 20.19$, $p < 0.001$, $d = 0.272$).

iii. Belief change is commensurate with change in prevalence estimate

We ran a linear mixed-effects model predicting belief change with condition and change in prevalence estimate as fixed effects and random intercepts per participant and item. This model revealed significant main effects of condition ($\beta = 1.75$, $t = 5.21$, $p < 0.001$) and change in prevalence estimate ($\beta = 0.14$, $t = 8.15$, $p < 0.001$). The interaction between condition and prevalence change was not significant ($p = 0.25$). This model predicted 13.5% of the variance (conditional R^2).

iv. Belief change is dependent on initial certainty

We fit a linear mixed-effects model with standardized certainty and prevalence condition as fixed effects and random intercepts per participant and item. The model revealed main effects of both scaled certainty ($\beta = 1.51$, $t = 7.49$, $p < .001$) and prevalence condition ($\beta = 5.49$, $t = 20.54$, $p < .001$), as well as a significant interaction ($\beta = -2.87$, $t = -10.66$, $p < .001$).

We replicated our predicted effect of certainty with an additional linear mixed-effects model using change in prevalence estimate as a continuous predictor instead of the dichotomous condition variable. This model had an otherwise identical structure to the first. Again, main effects of standardized certainty ($\beta = 0.41$, $t = 2.76$, $p < .01$) and change in prevalence estimate ($\beta = 0.18$, $t = 26.64$, $p < .001$) were significant. Crucially, the same significant interaction was observed ($\beta = -1.98$, $t = -15.10$, $p < .001$).

A confound explains the unexpected positive relationship between certainty and belief change in the Control condition. Estimates of prevalence differed more from participant's own initial beliefs when those beliefs were more certain. As a result of this pattern, the data shown in the Control condition differed more from participant's own beliefs under conditions of high certainty, potentially leading to higher belief change. To control for this confound, we added the raw difference between initial prevalence estimate and initial belief as a fixed effect to the previous model, and used it to predict belief change in the Control condition. With the addition of the confound to the model, the main effect of scaled certainty disappeared ($p = 0.14$). The model also revealed a significant effect of prevalence change ($\beta = 0.23$, $t = 14.30$, $p < .001$), a significant effect of the difference between initial prevalence estimate and initial belief ($\beta = 5.15$, $t = 26.52$, $p < .001$), and a significant, negative interaction between certainty and prevalence change ($\beta = -1.92$, $t = -11.34$, $p < .001$).

D. Evidence Against the Effect of Demand Characteristics in Experiment 2

Analyses of participants' open-ended responses about their perceived purpose of the study support the idea that demand characteristics are not a primary driver of the results in Experiment 2. First, many participants' responses suggested that they believed the memory cover task (e.g. "The ability of someone to remember a surprising result."; "how distraction from random questions affects short term memory over an extended time frame"). Second, we sorted participants into groups based on whether they gave any indication that they suspected the study had something to do with their beliefs being influenced by the prevalence data (e.g. "Social influence on people's endorsement of or belief in different ideas"; N = 68) or did not (N = 134). A linear mixed-effects model with prevalence condition and mention of social influence in the open-ended response as fixed effects and random intercepts per participant and item found no significant effect of this awareness ($p = 0.13$) and no significant interaction ($p = 0.64$). Belief change was the same between the two groups, suggesting that even explicit awareness of the intended purpose of the study did not affect behavior.

E. Alternate Figure: Belief Change is Dependent on Initial Certainty

Mean belief change vs. mean initial belief for each item, by condition. Lines represent the fit from linear regression for each condition, with 95% confidence intervals.

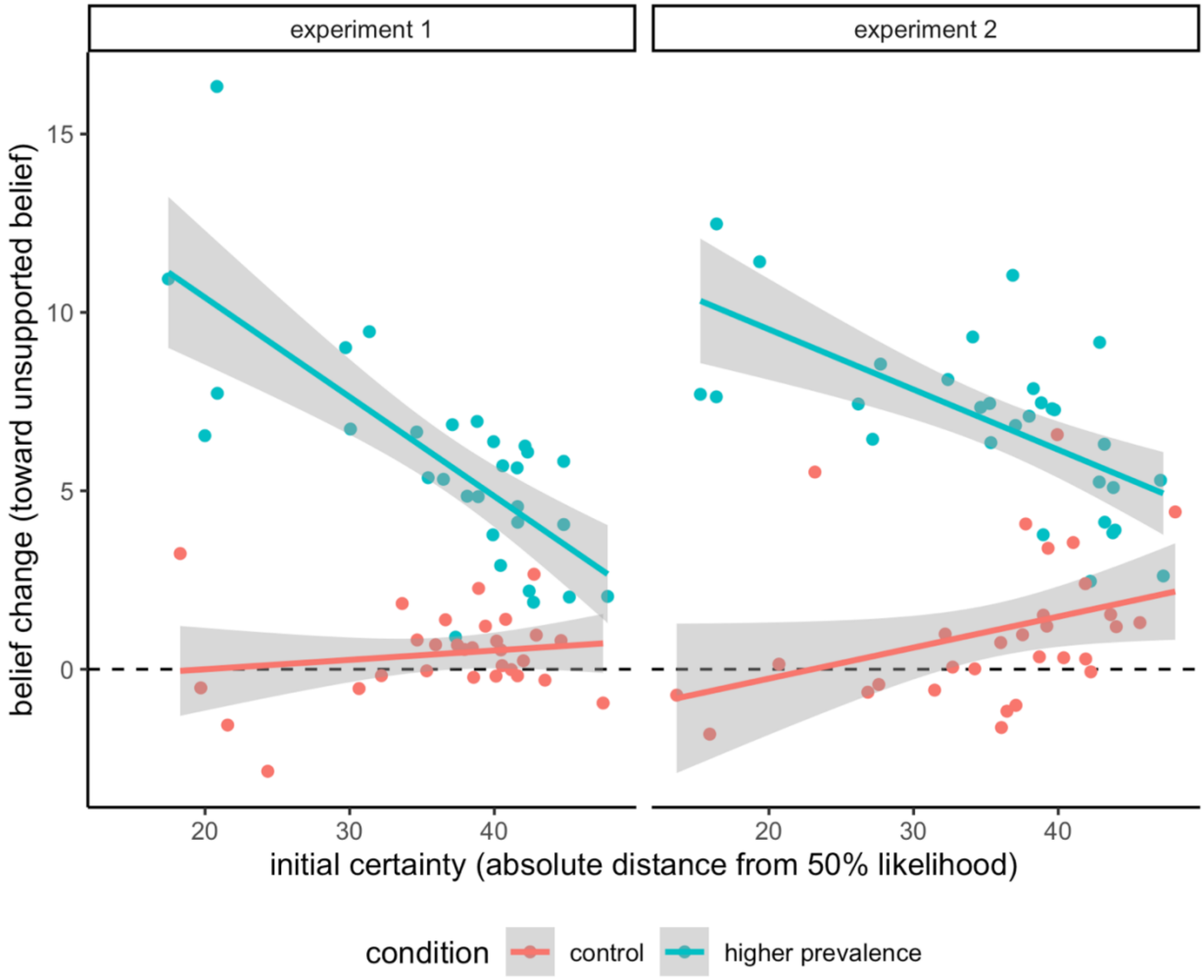

Supplement: Supplementary file 1 [file opmi-06-77-s001.pdf]
